# Fc glycans of therapeutic antibodies as critical quality attributes

**DOI:** 10.1093/glycob/cwv065

**Published:** 2015-08-11

**Authors:** Dietmar Reusch, Max L Tejada

**Affiliations:** 2 Pharma Biotech DevelopmentPenzberg, Roche Diagnostics GmbH, Penzberg 82377, Germany; 3 Biological Technologies, Genentech, CA 94080, USA

**Keywords:** biologic activity, critical quality attribute, Fc glycans, immunogenicity, therapeutic antibody

## Abstract

Critical quality attributes (CQA) are physical, chemical, biological or microbiological properties or characteristics that must be within an appropriate limit, range or distribution to ensure the desired product quality, safety and efficacy. For monoclonal antibody therapeutics that rely on fraction crystalizable (Fc)-mediated effector function for their clinical activity, the terminal sugars of Fc glycans have been shown to be critical for safety or efficacy. Different glycosylation variants have also been shown to influence the pharmacodynamic and pharmacokinetic behavior while other Fc glycan structural elements may be involved in adverse immune reactions. This review focuses on the role of Fc glycans as CQAs. Fc glycan information from the published literature is summarized and evaluated for impact on patient safety, immunogenicity, bioactivity and pharmacodynamics/pharmacokinetics.

## Introduction

Therapeutic monoclonal antibodies recognizing cell surface expressed antigens, can engage Fcγ receptors (FcγR) on effector cells (monocytes, macrophages, natural killer (NK) cells, neutrophils, eosinophils and dendritic cells), or bind to Complement 1q (C1q), and elicit immune effector functions such as antibody-dependent cellular phagocytosis (ADCP), antibody-dependent cell-mediated cytotoxicity (ADCC) and complement-dependent cytotoxicity (CDC) (Ravetch 1997). Additionally, direct apoptosis may be enhanced by effector cell dependent crosslinking (Ravetch 1997). The family of FcγR mediating these activities includes FcγRI, FcγRIIa, FcγRIIb, FcγRIIIa and FcγRIIIb [60–62]. These receptors differ in their antibody binding affinities [FcγRI binds with higher affinity to immunoglobulin G (IgG) than FcγRII and FcγRIII] and can be characterized as either activating (FcγRI, FcγRIIa and FcγRIII) or inhibitory (FcγRIIb).

**Fig. 1.**
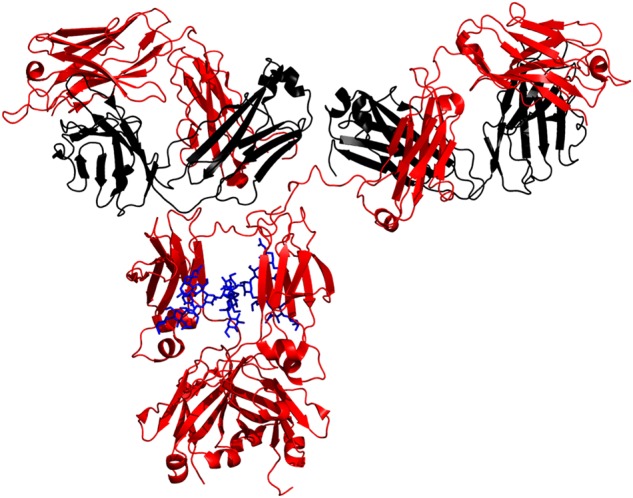
Ribbon model of an IgG1 (red: heavy chain; black: light chain; blue: glycans) (drawn with pyMol).

Antibodies (also commonly referred to as immunoglobulins, or IgG) consist of two heavy chains and two light chains. Each heavy chain contains three domains, CH1, CH2 and CH3 with a hinge region between CH1 and CH2. The heavy chain contains biantennary *N*-glycan structures linked to Asn297 in the CH2 domain (Jefferis 2009a). The two glycan chains on each of the CH2 domains are often different and contribute to the asymmetrical binding of the fraction crystalizable (Fc) to the Fc-receptors (Ferrara et al. 2006; Masuda et al. 2007; Mimura et al. 2007; Jefferis 2012). Fc glycosylation also stabilizes the Fc structure (Bowden et al. 2012). N-glycosylation is one of the most important post-translational modifications and often results in a remarkable heterogeneity of protein glycoforms (Jefferis 2005; Beck et al. 2008). Depending on the recombinant expression system, this may be a complex type, hybrid type or high-mannose type structure (Durocher and Butler 2009; Jefferis 2009a). The use of mammalian expression systems generally results in complex type biantennary oligosaccharides in the Fc portions. These glycans may have a core fucose and a bisecting *N*-acetylglucosamine. Moreover, they can vary in terminal galactose and sialic acid content (see Figure 1 and Table I) (Parekh et al. 1985; Jefferis 2005; Durocher and Butler 2009; Jefferis 2009a). In addition, some IgGs contain additional *N*-glycans in the variable regions of the fragment antigen binding (Fab) portion. Fab *N*-glycans have been described to differ from the oligosaccharides of the Fc region in that they are generally more highly galactosylated and are more extensively decorated with sialic acids. About 15–20% of IgG are glycosylated in the Fab part with no impact to structure (Jefferis 2005; Holland et al. 2006; Huang et al. 2006; Lim et al. 2008). However, Fab glycosylation is not within the scope of this review.

**Table I.** Glycostructures commonly found in the Fc portion of a therapeutic antibody

Name and composition	Classification	Structure	Exemplary relative abundance for a therapeutic antibody^a^
G0F [H3N4F1]	Complex, fucosylated	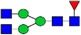	35.5
G1F [H4N4F1]	Complex, fucosylated	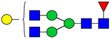	43.4
G2F [H5N4F1]	Complex, fucosylated	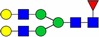	9.5
G1FS [H4N4F1S1]	Complex, fucosylated	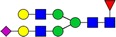	0.2
G2S1F [H5N4F1S1]	Complex, fucosylated	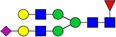	0.7
G2S2F [H5N4F1S2]	Complex, fucosylated	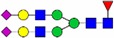	0.1
G0 [H3N4]	Complex, nonfucosylated	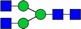	4.6
G1 [H4N4]	Complex, nonfucosylated	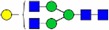	3.3
G2 [H5N4]	Complex, nonfucosylated	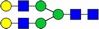	0.3
G0F-N [H3N3F1]	Hybrid (monoantennary)	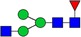	0.5
G0-N [H3N3]	Hybrid (monoantennary)	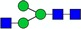	0.4
M5 [H5N2]	High mannose	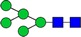	1.5
M6 [H6N2]	High mannose	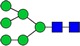	0.1

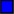
, *N*-acetylglucosamine; 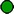, mannose; 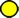, galactose; 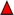, fucose; 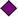, *N*-acetylneuraminic acid.

^a^Data from Reusch et al. (2015).

Where the mechanism of action (MoA) involves immune-mediated effector function, these activities can be greatly influenced by the Fc glycosylation pattern. For example, a high degree of galactosylation can promote activation of the complement system in vitro by increasing C1q binding and CDC and could have modest effects on ADCC (Hodoniczky et al. 2005; Nimmerjahn et al. 2007; Houde et al. 2010). It is also well known that decreases in core-fucose levels lead to a pronounced increase in ADCC via increased affinity of IgG1 for FcγRIIIa on immune cells (Okazaki et al. 2004; Jiang et al. 2011). Recent findings indicate that the lack of core fucose also promotes ADCP mediated by FcγRIIIa-positive monocytes and macrophages (Golay et al. 2013; Herter et al. 2014). Differences in glycosylation patterns can also lead to differences in the pharmacodynamic and pharmacokinetic behavior (Newkirk et al. 1996; Goetze et al. 2011). In addition, IgG glycan structural elements such as α1,3-bound galactose and *N*-glycolylneuraminic acid (NGNA) may be involved in adverse immune reactions (Zhu and Hurst 2002; Chung et al. 2008).

In some cases, a correlation has been established between FcγRIIIa phenotype and the clinical response, suggesting that ADCC activity could contribute to clinical efficacy (Cartron et al. 2002; Musolino et al. 2008; Bibeau et al. 2009). These results prompted the idea to use glycoengineering as a means of improving efficacy (Beck et al. 2008). Glycoengineering is a process used to manufacture antibodies lacking core fucose, which results in significantly higher binding affinity of human IgG1 antibodies to human FcγRIIIa, and consequently greatly enhanced ADCC activity. Shinkawa et al. (2003) showed a 50-fold increase in ADCC by IgG1s containing a high proportion of afucosylated Fc glycans. Highly afucosylated antibodies can be generated by modifying the oligosaccharide biosynthesis activities in various ways. For example, overexpression of *N-acetylglucosamine-transferase III* (GnTIII) in the Golgi apparatus of the production cell line generates bisected oligosaccharide structures associated with the Fc constant region of the antibody and suppresses fucosylation. In such expression systems, the level of GnTIII expression correlates with the generation of afucosylated IgG1 glycoforms and resulting enhanced ADCC activity (Ferrara et al. 2006). Highly afucosylated antibodies can also be produced using a fucosyltransferase-deficient producer Chinese hamster ovary (CHO) cell line. In this technology, both FUT8 alleles are disrupted by homologous recombination, resulting in completely afucosylated recombinant IgGs (Mori et al. 2004; Matsumiya et al. 2007).

A critical quality attribute (CQA) is defined as a physical, chemical, biological or microbiological property or characteristic that should be within an appropriate limit, range or distribution to ensure the desired product quality, safety and efficacy (Shinkawa et al. 2003). Identification of CQAs is a component of the quality by design (QbD) approach to product development, along with identification of the process parameters that affect these CQAs, the design and control of manufacturing via in-process and quality control testing of critical quality and performance attributes of raw and in-process materials and processes that ensure manufacturing consistency. When the mode of action of an antibody involves effector function, glycosylation represents one of the main sources of heterogeneity with potential impact to safety and efficacy, making it a critical manufacturing parameter to monitor. Therefore, a thorough characterization of carbohydrate content, the structure of the carbohydrate chain, the oligosaccharides and glycosylation sites present on the antibody is critical (Shinkawa et al. 2003).

This review focuses on product-specific clinical and nonclinical information, analytical and biological characterization, general and platform antibody knowledge and published literature.

## Results

### CQAs

The Annex to ICH Q8 defines CQAs as physical, chemical, biological or microbiological properties or characteristics that should be within an appropriate limit, range or distribution to ensure the desired product quality, safety/immunogenicity, efficacy and pharmacodynamics/pharmacokinetics (Table II).

**Table II.** Impact of Fc glycans on safety/immunogenicity, biologic activity/efficacy and clearance (PK/PD)

Glycan species	Safety/immunogenicity	Biologic activity/efficacy	Clearance (PK/PD)
Galactose	Unknown	+	Unknown
α1,3-galactose	−−	Unknown	Unknown
Fucose	(−)	++	Unknown
Bisecting GlcNac	(−)	+	Unknown
High mannose	Unknown	+	−−
NANA	Unknown	(−)	+
NGNA	−−	(−)	+
β1,2-Xylose/α1,3-Fucose	−−	Unknown	Unknown
NGHC	Unknown	−	(−)

+ Positive impact; − negative impact; ++ high positive impact; −− high negative impact; (+/−) potential impact.

#### Safety/immunogenicity

The criticality assessment of a CQA with respect to safety and immunogenicity cannot be performed independent of qualitative and quantitative analysis of the various glycoforms and should take into account nonclinical, e.g. investigational new drug-enabling tox studies, and clinical experience.

Safety is assessed based on nonclinical observations most often from studies on primates such as cynomolgus monkey, as well as observed clinical adverse events (AEs). These AEs can be target-related or nontarget-related, i.e. “off-target effects”, anti-therapeutic antibody (ATA) related or ATA-independent.

AEs include infusion-related reactions (IRR), injection site reactions, hypersensitivity, anaphylaxis, rash, neutropenia, thrombocytopenia and so on. IRR may be linked to IgG Fc glycosylation, as certain glycoforms can mediate complement activation. Furthermore, Fc glycans that affect Fc effector functions may impact on safety either by increasing otherwise low cell killing potential or through secondary effects such as cytokine release triggered by the activation of effector cells (Baldo 2013).

#### Biologic activity (efficacy)

While the glycosylation pattern is not known to affect the interaction of an antibody with its target, it can greatly influence effector functions by modulating binding to FcγRγ on immune cells (Lund et al. 1995; Daeron 1997; Ravetch 1997; Gerber and Mosser 2001; Ravetch and Bolland 2001; Sondermann et al. 2001; Radaev and Sun 2002; Nimmerjahn and Ravetch 2006). Therefore, it is important to consider the contribution of Fc effector function to the MoA as part of the CQA assessment. This holds true independent of the classification of therapeutic antibodies based on their putative mechanisms of action (Class I MoA: cell-bound antigen with depletion; Class II MoA: cell-bound antigen with functional blocking; Class III MoA: soluble antigen with blocking) (Jiang et al. 2011).

#### Clearance (PK/PD)

The neonatal Fc receptor (FcRn) plays a role in adult salvage of IgG. FcRn in the acidic endosomes bind to IgG internalized through transcytosis. The IgG is recycled to the cell surface and is released at the pH of blood (so it is prevented from lysosomal degradation). While aglycosylation has profound impacts to effector function, the interaction of IgG-Fc with FcRn is believed to be independent of Fc glycosylation (Jefferis 2012). Changes of the levels of the different glycoforms of the antibody as a component of circulation time are interpreted as arising from differences in clearance rates. In principle, this could be due to differences in binding to the FcRn (Ghetie and Ward 2000; Akilesh et al. 2007; Roopenian and Akilesh 2007; Kuo et al. 2009; Tesar and Bjorkman 2010) or to differences in the clearing rate mediated by C-type lectins such as dendritic cell-specific intercellular adhesion molecule-3-grabbing nonintegrin (DC-SIGN) or nonclassical Fc-binding receptors including mannose-binding lectin 2 (MBL2), Dectin 1 and the macrophage mannose receptor (MMR) (Wileman et al. 1986; Weis et al. 1998; Dong et al. 1999; Lee et al. 2002; Allavena et al. 2004).

In general, the influence of Fc glycans on pharmacokinetics/pharmacodynamics (PK/PD) is not well understood and the literature concerning the effects of Fc glycosylation variability on PK properties is ambiguous (Deng et al. 2012). While some reports show no impact to PK for different glycospecies (Harris 2005; Huang et al. 2006; Jones et al. 2007; Millward et al. 2008; Chen et al. 2009), others report changes in PK (Wright and Morrison 1994; Newkirk et al. 1996; Kanda et al. 2007).

In contrast, Leabman et al. performed a comprehensive analysis regarding the impact of glycosylation on PK in cynomolgous monkey. This assessment includes data from aglycosylated antibodies generated via engineering (N297A, N297G) or by production in *Escherichia coli*, an antibody with a (L234A/L235A) LALA mutation as well as glycoengineered antibodies. The antibodies targeted different antigen types, including highly expressed multi-transmembrane receptors, soluble cytokines, cell surface proteins and ligands. The results of this comprehensive study demonstrated that antibodies with differences in glycosylation that significantly alter FcγRIIIa binding show no differences in PK (Leabman et al. 2013). The inclusion of a broad range of antibody targets in this study increases the likelihood that these findings are broadly applicable. Several studies have shown that glycoproteins, i.e. IgG-Fc fusion proteins with terminal *N*-acetylglucosamine residues, are thought to be cleared by means of the mannose receptor. However, this clearance was mediated by *N*-acetylglucosamine in the receptor region and the Fc glycan ratio did not change (Jones et al. 2007; Keck et al. 2008).

Fc-glycans may not be accessible to asialoglycoprotein or mannose receptors that could mediate antibody clearance. Tao and Morrison (1989) showed that the serum half-life of aglycosylated IgG-Gln [IgG1 with one *N*-acetyl-glucosamine (GlcNAc)] in mice remains the same as for wild-type IgG1. However, Dong et al. (1999) found an increase in binding and uptake of agalactosyl IgG by mannose receptor on macrophages and dendritic cells.

### Impact of Fc glycans

#### α1,3-Galactose

IgG glycan structural elements such as α1,3-bound galactose may be involved in adverse immune reactions. In humans, anti-α1,3-galactose IgG constitutes as much as 1% of circulating IgG. This glycovariant is unique among endogenous human antibodies because of its atypically high concentration in serum (30–100 µg/mL) and its presence in all humans (Galili et al. 1984).

Therapeutic monoclonal antibodies produced in murine myeloid cell lines like SP2/0 or NSO contain α1,3-gal structures on their Fc glycans (Larsen et al. 1989; Sheeley et al. 1997; Chung et al. 2008). It is generally accepted that CHO cells lack the biosynthetic machinery to synthesize glycoproteins with α1,3-gal moieties (Jenkins et al. 1996). Bosques et al. determined that CHO 1,3-α-galactosyltransferase-1 is active and detected α1,3-gal on two of their products; however, these products were CTLA4-Fc fusion proteins, and the α1,3-gal was on the CTLA4 portion. The authors indicated that this may be a phenomenon resulting during single cell cloning of the production cell line, and that this does not occur in all CHO cells (Bosques et al. 2010).

There are several reports discussing the role of α1,3-gal epitope in xenotransplantation and on glycoproteins (Borrebaeck et al. 1993; Galili 2001; Deglon et al. 2003). The most relevant case is that of cetuximab, a chimeric IgG1 monoclonal antibody (mAb) against the epidermal growth factor receptor that is approved for use in colorectal cancer and squamous-cell carcinoma of the head and neck. While cetuximab is glycosylated on both its Fc and Fab domains, the α1,3-gal structures are found only on the Fab portion (Qian et al. 2007). This may be related to the sequence in the CDR and the fact that cetuximab is produced using SP2/0 cells.

Chung et al. reported a high prevalence of hypersensitivity reactions to cetuximab in some regions of the United States. In most of the subjects who exhibited a hypersensitivity reaction, IgE antibodies specific for the galactose-α-1,3-galactose on cetuximab were present in serum prior to therapy (Chung et al. 2008).

In contrast, van Lammerts et al. reported that anti-α1,3-gal IgE from allergic patients do not bind α1,3-galactosylated glycans on intact therapeutic antibody Fc domains. The authors showed that cetuximab was bound by α-Gal-specific IgE antibodies in the serum of patients and that this binding is restricted to the Fab domain. Cetuximab Fc domains were not bound even though they contained detectable amounts of α1,3-gal. This is believed to be due to the inaccessibility of the α-Gal moiety in the Fc domain, since binding is observed due to glycan exposure following proteolytic digest. The authors observed an increased affinity of α1,3-gal IgE antibodies for glycostructures on the Fab domain bearing two α1,3-gal moieties suggesting that therapeutic mAbs produced in rodent cell lines, and only glycosylated in their Fc domains, are not recognized by α1,3-gal-specific IgE antibodies (Lammerts van Bueren et al. 2011). In conclusion, based on the literature, α1,3-gal in Fc-glycans may be a CQA concerning safety/immunogenicity. It should be closely monitored if Sp2/0 or NS0 cells are used for production of therapeutic antibodies.

#### β1,2-xylose and α1,3-fucose

Plants are attractive hosts for the manufacture of recombinant protein therapeutics as they are relatively inexpensive systems that can be readily scaled up. However, plant-derived monoclonal antibodies contain complex *N*-glycans containing β(1,2)-xylose and α1,3-fucose residues not present in humans and are therefore regarded as “carbohydrate cross-reactive determinants” in IgE from sera of allergic patients (Tekoah et al. 2004). The glycan-specific IgE show 23% prevalence and have been found at levels up to 71% in individuals with multiple pollen sensitivity. However, the clinical relevance of these reactive IgE remains unclear. Strasser et al. used RNAi to successfully silence XylT, the gene coding for β1,2-xylotransferase, in tomato plants and demonstrated a patient-specific reduction in IgE reactivity in their studies (Paulus et al. 2011). *Arabidopsis thaliana* knockouts of the XylT (encoding β1,2-xylotransferase) and FucT (encoding α1,3 fucosyl transferase) genes have also been generated that are viable with no obvious phenotype. This represents a path forward in the production of protein biotherapeutics with human type N-glycosylation (Strasser et al. 2004). In conclusion if plants are used to produce therapeutic antibodies and the transferases are not silenced, β1,2-xylose and α1,3-fucose should be taken into account as CQAs.

#### N-glycolylneuraminic acid

Glycans of therapeutic proteins that are produced in SP2/0, NSO and, to a much lesser extent, in CHO cell lines are often modified with the non-human sialic acid *N*-glycolylneuraminic acid (Neu5Gc; NGNA) (Hokke et al. 1990; Noguchi et al. 1996). Humans do not synthesize NGNA as the gene encoding CMP-*N*-acetylneuraminic acid hydroxylase, which produces CMP-NGNA from CMP-N-acetylneuraminic acid (NANA), is mutated (Varki 2007). It was previously thought that healthy humans did not react to NGNA; however, more recent findings suggest that all humans have anti-NGNA antibodies, some at levels up to 0.1–0.2% of circulating IgG. This may be due to dietary exposure, and the subsequent incorporation of NGNA into human proteins (Tangvoranuntakul et al. 2003; Padler-Karavani et al. 2008). Padler-Karavani et al. (2008) suggested that the ongoing antigen–antibody reaction may generate chronic inflammation, possibly contributing to the high frequency of diet-related carcinoma and other diseases in humans.

It has been demonstrated that many humans produce antibodies against oligosaccharide structures with terminal NGNA (Zhu and Hurst 2002). The potential impact of NGNA was evaluated by Durocher and Butler (2009) who indicated that this immunogenicity can reduce efficacy due to rapid clearance of the biotherapeutic, or alternatively, prevent drug re-administration due to an undesirable immune response. As these ATAs represent the first obstacle to xenotransplantation in humans, they are clinically significant from a safety perspective (Milland and Sandrin 2006; Higgins 2010).

Biotherapeutics produced in human cell lines may also become contaminated with NGNA that is incorporated from animal-derived culture medium materials (Ghaderi et al. 2010). NGNA in cells is recycled in lysosomes and incorporated into glycoproteins (Bardor et al. 2005).

In conclusion, NGNA on Fc-glycans is potentially immunogenic and for this reason is most probably a CQA.

#### Terminal sialic acid

The anti-inflammatory activity of immunoglobulin has repeatedly been linked to Fc sialylation, which may lead to decreased ADCC activity and, in some cases, even affect target binding (Fukushima and Takasaki 1993; Kaneko et al. 2006; Scallon et al. 2007; Anthony, Nimmerjahn, et al. 2008; Anthony, Wermeling et al. 2008; Nimmerjahn and Ravetch 2008). In general, the amount of sialylated Fc glycans of therapeutic antibodies is very low (Jefferis 2006).

In conclusion if there is a significant amount of sialylated glycans, and ADCC is part of the MoA of the therapeutic antibody, the influence of sialylation on FcγRIIIa binding and ADCC should be determined in vitro as part of the CQA assessment.

#### α1,6-core fucose

Changes in the levels of afucosylated glycostructures on therapeutic antibodies may be of concern from a safety perspective. Toxicity due to off-target binding may also be a concern for antibodies with higher ADCC. Jiang et al. indicate that increased afucosylation leading to increased effector function is a potential safety concern for monoclonal antibodies with moderate or low effector function (Jiang et al. 2011).

Increased crosslinking of activating FcγRs due to higher levels of afucose may also be a safety concern due to the additional release of proinflammatory cytokines such as tumor necrosis factor (TNF)-α and IFNγ.

The lack of core fucose (i.e. the α1,6-linked fucose on the GlcNAc residue involved in the amide bond with the asparagine of the N-glycosylation site) on the carbohydrate moiety linked to the Fc region of an IgG molecule leads to a pronounced increase in ADCC via increased affinity for the FcγRIIIa expressed on immune cells such as NK cells and macrophages (Rothman et al. 1989; Umana et al. 1999; Shields et al. 2002; Niwa et al. 2004; Okazaki et al. 2004; Yamane-Ohnuki et al. 2004; Peipp et al. 2008; Chung et al. 2012). Additionally, the absence of core fucose leads to increased Fc-dependent binding to FcγRIII positive nonclassical/intermediate monocytes and macrophages, which translates into increased ADCP (Herter et al. 2014). In contrast, binding to FcγRI and II-positive and FcγRIII-negative classical monocytes, as well as ADCP-mediated by these cells, remain unchanged, irrespective of fucosylation levels (Herter et al. 2014).

While afucosylation increases ADCP in glycoengineered antibodies, the impact on ADCP due to small variations in afucosylation levels resulting from standard CHO-based manufacturing processes remains to be assessed. Finally, increased afucosylation has not been demonstrated to have any impact on CDC activity.

Studies by Junttila et al. highlighted the impact of afucosylation on pharmacological properties and half-life of an IgG1, relative to a wild-type control. In these studies, afucosylated trastuzumab displayed moderately altered pharmacokinetic dispositions compared with trastuzumab, slightly faster elimination from the circulation, and a modest reduction in half-life (Junttila et al. 2010). The authors hypothesized that the faster clearance may result from the differential bio-distribution that leads to the enrichment of antibodies to immune effector cell-rich organs due to the increased FcγRIII affinity. In conclusion concerning the scientific literature there is no clear evidence that afucosylated Fc glycans might be of concern for safety.

There is clear evidence that afucosylated Fc glycans increase ADCC and if ADCC is part of the MoA, afucosylated Fc-glycans are always likely to be a CQA concerning efficacy.

#### Bisecting GlcNAc

CHO cells lack the gene encoding GnTIII. Therefore antibodies produced in CHO cells lack detectable bisecting GlcNAc glycostructures (Durocher and Butler 2009). GnTIII overexpressed in antibody-producing cells catalyzes the addition of GlcNAc to N-linked oligosaccharides. The addition of bisecting GlcNAc inhibits core-fucosylation and conversion of hybrid to complex glycans (Ferrara et al. 2006).

In general, glycostructures containing bisecting GlcNAc are abundant in human polyclonal IgG and are not a considered a safety concern (Jefferis 2009a). Recently, Ritamo et al. (2013) showed that 7% of the Fc-glycans on intravenous IgG (IVIG) contain glycan structures with bisecting GlcNAc (analyzed at the glycopeptide level) (Ritamo et al. 2013). Therefore, it is unlikely that ATAs are produced against the bisecting GlcNAc epitope. In summary, bisecting GlcNac structures are not a safety issue.

#### Galactose

Highly galactosylated glycostructures on therapeutic antibodies may be of concern as they increase in vitro C1q-binding and CDC activity (Boyd et al. 1995; Tsuchiya et al. 1989). Increased galactosylation of CHO-derived antibodies has been shown to increase FcγRII and FcγRIIIa binding as measured using surface plasmon resonance (Ritamo et al. 2013). Additionally, it has been demonstrated that the increased galactosylation enhances ADCC activity (Jiang et al. 2011). However, the impact of galactosylation is subtle compared with the impact of afucosylation (Ritamo et al. 2013).

Several reports indicate that a high degree of galactosylation promotes increased binding to C1q leading to enhanced activation of the complement system and CDC activity (Hodoniczky et al. 2005; Jefferis 2009a; Nimmerjahn et al. 2007). Similar effects have been observed for the glycoengineered therapeutic antibody obinutuzumab in vitro*,* although CDC is not considered to be a relevant in vivo activity. Several reports state that the extent of terminal galactosylation does not affect ADCC in standard IgG1 antibodies (Boyd et al. 1995; Shinkawa et al. 2003; Hodoniczky et al. 2005). Another report found that terminal galactosylation has a positive effect on FcγRIIIa binding (Houde et al. 2010). We have recently demonstrated enhanced ADCC upon enzymatic hypergalactosylation of four different monoclonal antibodies produced using standard CHO manufacturing processes (Tejada et al. submitted; Thomann et al. 2015). We also showed that elevated levels of terminal galactose have no effect on ADCC of two different glycoengineered therapeutic antibodies (Thomann et al. 2015), indicating that the extent to which terminal galactose modulates ADCC depends on the background level of afucosylation. Lastly, we quantitatively compared the effects of galactosylation and afucosylation in the context of glycan heterogeneity to demonstrate that while galactose can influence ADCC activity, afucosylation remains the primary driver of this activity.

Karsten et al. (2012) recently reported an anti-inflammatory activity of IgG1 mediated by Fc galactosylation and association of FcγRIIb and dectin-1. The authors concluded that high *N*-glycan galactosylation of IgG1 molecules promotes cooperative signaling of the FcγRIIb with dectin-1, resulting in an inhibitory signaling pathway that blocks proinflammatory effector functions.

In conclusion if CDC is part of the MoA for a therapeutic antibody the impact of galactosylation levels reflecting the manufacturing experience should be considered as it relates to efficacy during CQA assessment. If ADCC is part of the MoA of the therapeutic antibody, the impact of galactosylation should be determined by in vitro studies as part of CQA assessment.

#### High mannose glycostructures

No data or direct evidence in the literature supports the immunogenicity of high mannose glycan structures (Pacis et al. 2011). Studies by Zhou et al. reported enhanced FcγRIIIa binding and ADCC in antibodies with oligomannose-type glycans generated using kifunensine (Yu et al. 2012). Kanda et al. (2007) generated core fucose-lacking human IgG1 antibodies with three different N-linked Fc oligosaccharides, namely, a high-mannose, hybrid and complex type, using the same producing clone. They demonstrated that the mannosylated glycostructures could induce ADCC via FcγRIIIa binding but to a lesser extent than the hybrid and complex structures. The same holds true for CDC and C1q binding. More recently, Yu et al. (2012) confirmed that high mannose glycoforms exhibited higher FcγRIIIa binding and ADCC activity as well as decreased C1q binding and CDC activity.

In general, core fucose is absent in the highly mannosylated glycoforms generated. Therefore, it remains unclear if the observed increases in FcγRIIIa binding and ADCC activity are due to the presence of multiple mannose moieties, or the absence of core fucose. The reduced C1q binding and CDC activity observed in these studies may also reflect the lack of terminal galactose in these highly mannosylated forms.

Recent studies show clear evidence for selective clearance of oligomannose forms of IgG1 and IgG2 most likely via a mannose receptor-mediated mechanism (Goetze et al. 2011; Alessandri et al. 2012; Yu et al. 2012). Goetze et al. (2011) used IgG1 and IgG2 in human studies to demonstrate that Man5 isoforms were preferentially removed from circulation, independent of the route of administration. In conclusion if there is a significant amount of mannosylated glycans and if ADCC is part of the mode of action of a therapeutic antibody, mannosylation may be a CQA concerning efficacy. Additionally, there is clear evidence that oligomannose bearing IgGs are faster cleared than others and therefore may be a CQA concerning PK/PD.

#### Nonglycosylated heavy chain

Jung et al. (2011) concluded that the unmasking of the region around Asn297 does not result in the formation of a neo-epitope. At present, no adverse immunogenicity effects associated with high levels of nonglycosylated heavy chain (NGHC) have been reported in any clinical study. Furthermore, antibodies with high levels of NGHC have significantly reduced bioactivity and effector function. Therefore, NGHC is likely not a concern from a safety or immunogenicity perspective. Aglycosylation of Asn297 completely abolishes binding to FcγRIII and reduces the binding affinity to C1q 10-fold (Tao and Morrison 1989; Mimura et al. 2000). The reduced binding affinity of aglycosylated antibodies abrogates both ADCC and CDC activities (Tao and Morrison 1989; Lund et al. 1996; Sazinsky et al. 2008; Jefferis 2009b; Jung et al. 2010, 2011). Ha et al. isolated hemi-glycosylated mAb (mAb with only one heavy chain being glycosylated and the other one being aglycosylated). It was separated from fully glycosylated and aglycosylated forms using cation-exchange chromatography (Ha et al. 2011).These studies demonstrated that the hemi-glycosylated mAb had decreased binding to all FcγR including both activating and inhibiting receptors. The hemi-glycosylated form also showed decreased ADCC using peripheral blood mononuclear cells as effector cells. The studies also showed a modest, but statistically significant, decrease in C1q binding for the hemi-glycosylated mAb. Shatz et al. (2013) generated heterodimers with different glycan structures and showed that removal of fucose from only one chain of a fully glycosylated heterodimer is sufficient to fully restore ADCC activity relative to a wild-type molecule. However, reduced potencies were observed for hemi-glycosylated or hemi-glycosylated-hemi-afucosylated heterodimers, relative to wild-type or a fully glycosylated heterodimer. These studies showed that afucosylation has a significant impact on the ADCC activity of the hemi-glycosylated species; however, the impact of afucosylation is far greater when both heavy chains are glycosylated. Aglycosylated antibodies have been shown to exhibit conformational changes, decreased thermal stability, increased tendency to aggregate and loss of effector functions (Ghirlando et al. 1999; Hristodorov et al. 2013). Evidence regarding the impact of aglycosylation on PK is inconclusive as there are studies demonstrating decreased and normal half-life. For example, comparisons of recombinant chimeric or mouse monoclonal antibodies against N297A mutant versions in mouse or rat show reduced half-lives for the mutant aglycosylated antibodies compared with their wild-type counterparts (Wawrzynczak et al. 1989, 1992).

In contrast, Hristodorov et al. (2013) generated six different wild-type antibodies and compared them to aglycosylated versions, generated via N297A mutation. The aglycosylated antibodies were found to be less stable and displayed a propensity to aggregate when subjected to low pH reflecting viral inactivation conditions common in the antibody manufacturing process. The PK properties of both wild-type and aglycosylated antibodies were determined to be nearly identical in rat studies. Tao and Morrison (1989) used a chimeric mouse-human IgG and found a shorter half-life for aglycosylated IgG3 compared with that of wild-type IgG3 in mice, while no change was observed for aglycosylated IgG1 compared with wild-type IgG1 (Tao and Morrison 1989). Jung et al. (2011) state that aglycosylated antibodies display PK that are at least comparable to that of “normal” IgG therapeutics.

In conclusion, the literature is not quite clear here and if there is a significant amount of NGHC the influence on product quality should be assessed by in vitro studies to decide if it is a CQA or not.

## Discussion

The QbD paradigm requires an in-depth understanding of (i) the mechanism(s) of action of the biotherapeutic, which are associated with the desired clinical activity, (ii) the properties (CQA) of the biotherapeutic that are associated with the desired clinical activity, (iii) the process parameters that impact the CQA and (iv) the control of the manufacturing process through a timely analysis and monitoring of the CQA, along with appropriate control of process components such as raw and in-process materials. This ensures that the product with the desired qualities is achieved consistently over time.

For mAb therapeutics specifically, glycosylation is one of the most relevant post-translational modifications in the manufacturing process. This results in remarkable heterogeneity of the antibody glycoforms. The diverse set of hosts used by industry in the manufacture of monoclonal antibodies also increases this glyco-heterogeneity, which can be critical to antibodies associated with MoAs such as CDC, ADCC and ADCP.

Different glycovariants such as high mannose, have been reported to reduce the serum half-life of therapeutic antibodies while other Fc glycan structural elements such as α1,3-bound galactose and NGNA may be involved in adverse immune reactions. Both should be considered in terms of efficacy and safety, respectively.

The intimate relationship established between Fc glycosylation of the mechanisms associated with the clinical activity of therapeutic antibodies makes the thorough analysis and characterization of the glycosylation-specific CQA critical to the appropriate assessment of the impact that changes observed in specific glycovariants may have on safety and/or efficacy. This information can appropriately inform manufacturing process development such that these processes are more finely adjusted to deliver the desired Fc glycosylation.

## Abbreviations

ADCC, antibody-dependent cellular cytotoxicity; ADCP, antibody-dependent cellular phagocytosis; AE, adverse events; ATA, anti-therapeutic antibody; CDC, complement dependent cytotoxicity; CHO, Chinese hamster ovary; CQA, critical quality attribute; C1q, Complement 1q; DC-SIGN, dendritic cell-specific intercellular adhesion molecule-3-grabbing nonintegrin; Fab, fragment antigen binding; Fc, fraction crystalizable; FcγR, Fcγ receptors; FcRn, neonatal Fc receptor; GlcNac, *N*-acetyl-glucosamine; GnTIII, *N*-acetylglucosamine-transferase III; IFN, interferon; IgG, immunoglobulin G; IRR, infusion-related reactions; MBL2, mannose-binding lectin 2; mAb, monoclonal antibody; MMR, macrophage mannose receptor; MoA, mechanism/mode of action; NGHC, nonglycosylated heavy chain; NGNA, *N*-glycolylneuraminic acid; NK cells, natural killer cells; PK/PD, pharmacokinetics/pharmacodynamics; QbD, quality by design; TNF, tumor necrosis factor.
